# Application of the Community Oral Health Indicator by Non-Dental Personnel and Its Contribution to Oral Healthcare

**DOI:** 10.1371/journal.pone.0039733

**Published:** 2012-07-27

**Authors:** Maria Vieira de Lima Saintrain, Anya Pimentel Gomes Fernandes Vieira

**Affiliations:** 1 School of Dentistry and Public Health Master Program, University of Fortaleza (UNIFOR), Fortaleza, Ceará, Brazil; 2 Oswaldo Cruz Foundation, Fortaleza, Ceará, Brazil; University of Toronto, Canada

## Abstract

**Objective:**

To validate the Community Oral Health Indicator-COHI by non-dental personnel.

**Methods:**

Risk assessment is an essential component in the decision-making process. Therefore, the COHI, an instrument to evaluate population oral health situation in a simple manner, was created. Community Health Agents (CHA) were trained to use the COHI (variables as number of teeth, presence of cavities, residual dental roots, oral lesions, etc.), while dentists for the COHI and DMFT. 60 individuals were examined, by CHA and DS, with these indicators in order to validate the use of COHI by non-dental personnel.

**Results:**

Dental and soft tissues problems were well spread among those individuals. People with and without soft tissue damage, as well as with and without use and/or need for prostheses were found in the sample, proving it to be a heterogeneous population for the evaluated factors and representing the real population. The results of examinations performed by dentists using the COHI and DMF-T/dmf-t presented strong agreement when comparing the two instruments. When COHI and DMFT were compared, the results showed a concordance of 0.86 for the number of present teeth, and 0.85 for the number of residual roots. Likewise, when analyzing the data comparing the use of the COHI by DS and CHA a high agreement level, specificity and sensitivity was found.

**Conclusion:**

The COHI has shown to be useful for detecting problems in oral health. Therefore, COHI may be used, after training, by non-dental personnel, contributing to the planning and organization of the community dental assistance.

## Introduction

Risk assessment is an essential component in the decision-making process for the prevention and management of dental caries, as well as for organizing services based on patient’s need. Along with the dramatic decline in caries prevalence during the past 30 years [1], the search for acceptable, accurate, and cost-effective strategies for identifying high-risk individuals has been intensified [2]. It is known that the implementation of public health policies that have the potential to reduce inequalities in oral health requires simplified actions, which should be universal in scope and involving social actors’ participation. Therefore, it is necessary to uptake appropriate technologies, used to improve work processes and management, known as process technologies [3].

Index/indicators of oral health are important tools in understanding population and individuals oral health risk, as well as in the organization of services. However, to date, despite the existence of specific instruments for use in dentistry, such as dmf-t [4], DMF-T [5], DMF-S [6], Gingival Index [7], Community Periodontal Index [8] and Loss of periodontal insertion [8], there are difficulties in their use in a continuous and systematic manner in a population basis, mainly due to the high cost and availability of dentists for this purpose. The use of health indicators by other professionals restricts this problem and has the potential to collect data capable of assessing the oral health of the population, enabling the organization of the services, including its demands.

For these reasons, an instrument designed to evaluate the oral health situation of the population in a simple manner, denominated Community Oral Health Indicator – COHI [9], was created. This instrument utilizes different health professionals (including non-dental personnel) for a simplified oral examination. This indicator, on top of having the ability to evaluate the oral health condition in an individual and collective basis, facilitates the working process of dentists in the community, contributing to the organization of dental assistance in a location. In this way, it is possible, without ignoring the collective actions of the dental service, to refer in a priority manner those individuals most in need of treatment. It is evident that this "new technology" provides insights for policy making and actions planning to promote oral health, including the organization of the health system demands, while respecting the principle of equity. Moreover, the planning and implementation of services (both preventive and curative) can be made in a more productive manner if the needs of the population are known [10].

Therefore, the aim of this study was to employ the Community Oral Health Index (COHI) by non-dental personnel (Community Health Agents-CHA). In the Brazilian National Health System, the CHA is a person from the community, with high school degree, who is properly trained to be the link between the community and the Health Care Unit. This professional was chosen to employ the COHI because home visits are already one of their daily activities (aiming to assess the population general health and their need for health care) and also because of their relative low cost for the health system.

## Methods

Initially, two CHA and two dentists (DS) were trained and calibrated to use the COHI. For this calibration, a trained, experienced dental examiner (co-author MVLS) acted as the gold standard. Kappa values of 0.8 were used as cut point for the calibration, meaning that the CHA and DS being trained were only considered calibrated when the intra and inter (against the gold standard) examiners kappa value were equal or above 0.8. Following the WHO guidelines, each CHA or dentist examined at least 20 individuals during the calibration process [11], in the occurrence of a p value below 0.8, the CHA or DS repeated the calibration process with another 10 subjects from the community.

**Figure 1:**
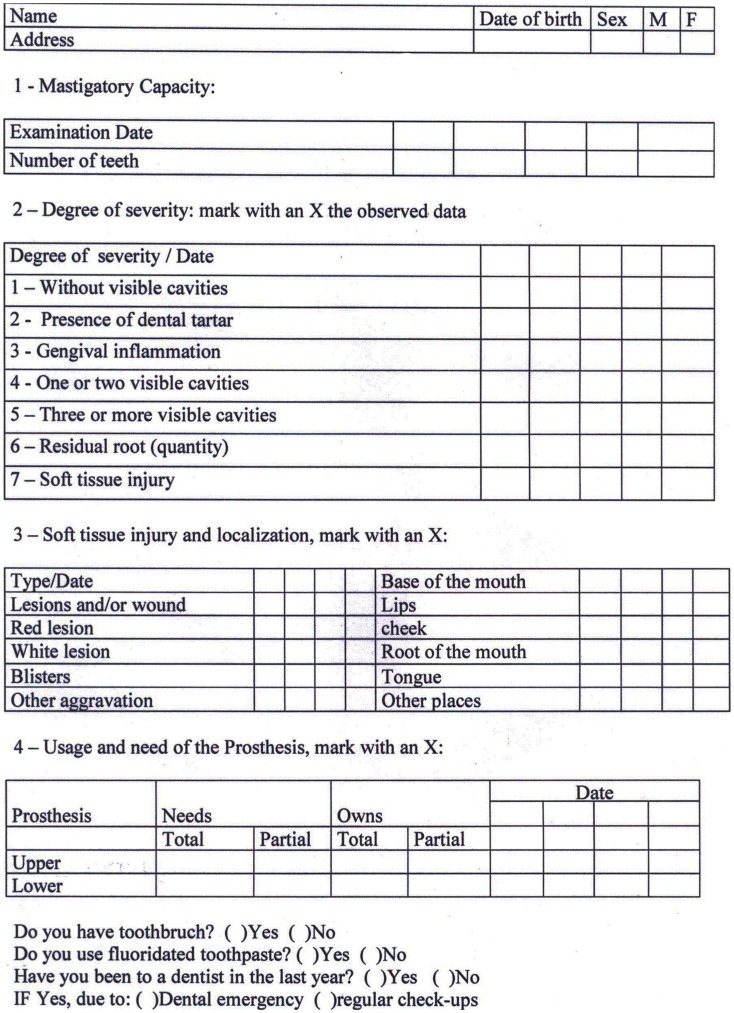
Community Oral Health Indicator – COHI.

This index checks the masticatory capacity by counting the number of teeth, the need for curative treatment by counting the visible teeth with cavities and residual root, the presence of soft tissues injuries, and the use and need of dental prosthesis. Additionally, this index possesses a list of clinical signs related to periodontal and dental cavities problems, as well as soft tissue injuries. This list of seven items (1-No dental cavity; 2-Tartar; 3-Gingival inflammation; 4-One or two dental cavities; 5-Three or more dental cavities; 6-Residual root; 7-Soft tissue injury) allows the prioritization of individuals in more severe need, whereas the 1 is related to lower priority of treatment and 7 to the highest priority (Figure 1).

The dentists were also trained and calibrated to use the DMF-T and dmf-t, which is the number of decayed, missing and filled teeth in the permanent and primary dentition [4]–[5]. For this, the same trained, experienced dental examiner (co-author MVLS) acted as a gold standard, and the WHO guidelines [11] for data reliability was used, which means that at least 20 individuals were examined by each dentist during his/hers calibration process. The DMF-T, developed by Klein and Palmer [5], and the dmf-t by Gruebbel [4] are widely used by the international scientific community in research and epidemiological surveys. However, despite having good sensitivity and specificity, these indexes can only be used by trained dentists. DMF-T expresses the number of permanent teeth attacked by cavities, with the average being the result of the sum of decayed, missing and filled teeth, divided up by the number of examined persons [12], while the dmf-t expresses the number of deciduous teeth (baby teeth) attacked by cavities, with the average being the result of the sum of decayed, missing (due to cavities) or decayed and filled teeth divided by the number of children examined [4].

Although the same training methodology was applied for both professional classes, the calibration occurred at separate times for the dentists and CHA. Initially, the COHI was presented and discussed, detailing their use and completion. For dentists, the DMF-T was also explained and discussed. In the second stage, the professionals were trained in order to recognize prevalent epidemiological problems in the oral cavity. With the help of *slides/data show,* photos with different diseases of the oral cavity were used to recognize healthy teeth, teeth with dental cavities, use and need of prosthesis and soft tissue injuries, including those arising from the use of a maladaptive prostheses, candidiasis and periodontal diseases. The third moment consisted of the training of professionals in the community, where they had the opportunity to work together (dentists separated from the CHA) to evaluate the oral cavity of patients on the premises of health facilities. The conflicting findings were discussed until a consensus was reached. The fourth training moment was the completion of the kappa test between the examiners and between them and the gold standard. The kappa test was used to assess the concordance of findings between the dentists on the use of DMF-T, dmf-t and COHI, and between dentists and CHA in relation to the COHI. This step was repeated until all the professionals had a value of agreement (kappa) greater than 0.8 with the gold standard (co-author MVLS), with oneself (intra-examiner calibration) and with other professionals (inter-examiner calibration).

After calibration, 60 residents of the city of Guaiúba-Brazil were examined by these professionals. It is important to note that even not being an epidemiological survey, the World Health Organization methods [11] for data collection, as described in their publication “Oral Health Survey-Basic Methods,” were utilized when appropriated. The CHA conducted the test using the COHI and the dentists (DS) using the COHI and DMFT/dmft. To avoid bias, DS and CHA were organized so that each could evaluate 30 research subjects. Thus, each resident was examined by two professionals (one DS and one CHA), where 15 were examined by DS 1 and ACS 1, 15 by DS 1 and ACS 2, 15 by DS 2 and 1 ACS, and finally, 15 by DS 2 and ACS 2. During data collection, examiners were unaware of each other findings, since the residents were examined separately by each professional. These examiners were in separate rooms for the evaluations, but the same conditions for test were utilized (*e.g.,* location, lighting and instruments). The volunteers were sitting in dental chairs under natural light. The survey was conducted with the help of a wooden spatula (tongue depressor). Being professionals, the examiners were duly dressed with personal protective equipment.

After collecting the data, it was digitized and organized in the statistical program Statistical Package for Social Science (SPSS) 15.0 (SPSS Co., Chicago, USA). The chi-square test, Spearman correlation, sensitivity, specificity, *kappa* and *weighted Cohen's kappa test* were used to compare the findings. The validation was performed in two phases: a) comparing results using the COHI and DMFT/dmft index by dentists, and b) comparing the findings of dentists and CHA using the COHI. Thus, dentists served as the gold standard for the CHA and DMFT as the gold standard for the COHI.

In order to compare the number of decayed teeth between COHI and DMFT/dmft a categorization was established for the DMFT/dmft in three levels of dental decay severity: without dental cavity, 1 or 2 cavities, and 3 or more cavities. This was necessary because DMFT/dmft is a continuous variable for dental decay, while the signs of dental decayed (observed as dental cavities) in the COHI is a categorical variable.

This study was submitted to the Research Ethics Committee at University of Fortaleza-Brazil and was approved (process No. 001/2007). A consent form was signed by all the participants, who were aware of the objectives and procedures. The participant also had guaranteed the confidentiality and freedom to withdraw consent at any stage of the research.

## Results

To validate the COHI, a total of 60 individuals in the municipality of Guaiúba-Brazil were concurrently examined by calibrated CHA and DS. The average age of respondents was 39.3 (SD±22.10) years, ranging from 6 to 87 years of age. Among those evaluated, 37 (62.0%) were female. The number of teeth in their mouth ranged from 0 to 30, the variation in the number of residual roots ranged from 0 to 13, while 0 through 28 teeth with dental decay were found in the oral cavities of examined individuals. Ten individuals were edentulous. The individuals were divided into children (up to 12 years old), adolescents (from 13 to 18 years old), adults (from 19 to 59 years) and elders (60 or older). It was noted, as it can be seen in table 1, that the dental and soft tissues problems were well spread among those categories, representing what is expected to be seen in the real population. Additionally, people with and without soft tissue damage, as well as with and without use and/or need for prostheses were found in the sample, proving it to be a heterogeneous population for the evaluated factors.

**Table 1:** Oral health characteristics of the population evaluated for the COHI validation.

	n (%)	Number of teeth (range)	DMFT/dmft (range)	Presence of Tartar	No cavity (count)	1 or 2 cavities (count)	3+ cavities (count)	Gingival inflammation (count)	Soft tissue injury (count)	Residual root (n^o^ individual – range of root per individual)
Children (up to 12 yrs)	7 (11,7)	23–27	0–5	1	2	3	2	0	0	0
Adolescents (13–18 yrs)	10 (16,7)	24–30	2–14	1	1	4	5	2	2	0
Adults (19–59 yrs)	31 (51,7)	0–30	0–32	18	11	9	11	7	3	8 (1–13)
Elders (60+ yrs)	12 (20)	0–22	16–32	6	6	3	3	3	5	6 (2–5)

Weighted kappa and spearman correlation were used to compare data between COHI and DMF-T evaluated by dentists regarding number of teeth and number of residuals roots. The results of examinations performed by dentists using the COHI and DMF-T/dmf-t presented strong agreement when comparing the two instruments. The agreement between the continuous variable were above 0.9 for both kappa and spearman tests. Chi-square and kappa were used to compare agreement between categorical variables (e.g., cavity categorization) when comparing COHI and DMF-T evaluated by dentists. For the agreement between categorized dental cavities, the results also show very significant values for the *kappa* and *chi-square* tests (Table 2).

**Table 2:** Comparative data between COHI and DMFT/dmft evaluated by dentists Guaiuba - Ceará, Brazil.

	n (%)	Number of teeth (range)	DMFT/dmft (range)	Presence of Tartar	No cavity (count)	1 or 2 cavities (count)	3+ cavities (count)	Gingival inflammation (count)	Soft tissue injury (count)	Residual root (n^o^ individual – range of root per individual)
Children (up to 12 yrs)	7 (11,7)	23–27	0–5	1	2	3	2	0	0	0
Adolescents (13–18 yrs)	10 (16,7)	24–30	2–14	1	1	4	5	2	2	0
Adults (19–59 yrs)	31 (51,7)	0–30	0–32	18	11	9	11	7	3	8 (1–13)
Elders (60+ yrs)	12 (20)	0–22	16–32	6	6	3	3	3	5	6 (2–5)

The weighted *kappa* test was used to assess agreement between dentists and CHA when using the COHI observing the number of present teeth, as well as the number of roots remaining in the oral cavity of individuals in the community. The results showed a concordance of 0.86 for the number of present teeth, and 0.85 for the number of residual roots.

Kappa, sensitivity and specificity tests were used to compare categorical data (e.g., presence of cavities, tartar, inflamed gums, soft tissue injury) when DS and CHA used COHI. When analyzing the data comparing the use of the COHI by DS and CHA a high agreement level, specificity and sensitivity of the results was found. It is important to know that no interviewee had lower partial denture (Table 3).

**Table 3:** Comparative data between COHI evaluated by dentists (DS_COHI_) and community health agents (CHA_COHI_) Guaiuba-Ceará, Brazil.

Concordance between continuous variables							
		Spearman correlation				Weighted Kappa	
Number of teeth in the oral cavity		r _s_ = 0.940 (p<0.001)				Kappa = 0.86 (p<0.001)	
Number of residual roots		r _s_ = 0.916 (p<0.001)				Kappa = 0.85 (p<0.001)	
Agreement between categorical variables*							
	**DS_COHI_**		**CHA_COHI_**	**Kappa**	**Sensitivity (%)**		**Specificity (%)**
Number of individualsWithout cavities	20		22	kappa = 0.74 (p<0.001)	83.3		96.4
Categorization cavities							
1 or 2 cavities	19		20	Kappa = 0.51 (p<0.001)	68.4		83.3
3 or more cavities	21		18	Kappa = 0.52 (p<0.001)	63.6		87.2
Presence of tartar	26		27	kappa = 0.77 (p<0.001)	88.4		88.6
Inflamed gums	12		9	kappa = 0.71 (p<0.001)	66.6		98
Soft tissue injury							
Type of injury: injuries or wounds	1		2	kappa = 0.66 (p<0.005)	100		98.3
Type of injury: White lesions	2		5	kappa = 0.55 (p<0.001)	100		94.9
Type of injury: blisters	2		2	kappa = 0.48 (p<0.001)	50		98.3
Type of injury: red lesions	6		6	kappa = 0.63 (p<0.005)	66.7		96.4
Location of injury: lips	1		2	kappa = 0.66 (p<0.005)	100		98.3
Location of injury: cheek	3		3	kappa = 0.65 (p<0.005)	66.6		98.3
Location of injury: palate	9		9	kappa = 0.88 (p<0.001)	90		98
Dental prosthesis							
Use prosthesis: total upper	13		12	kappa = 0.95 (p<0.001)	92.3		100
Use prosthesis: total lower	5		4	kappa = 0.88 (p<0.001)	80		100
Use prosthesis: partial upper	6		4	kappa = 0.82 (p<0.001)	83.3		98.2
Need prosthesis: total upper	10		10	kappa = 0.88 (p<0.001)	90		98
Need prosthesis: total lower	9		10	kappa = 0.94 (p<0.001)	100		98
Need prosthesis: partial upper	14		15	kappa = 0.86 (p<0.001)	92.9		95.7
Need prosthesis: partial lower	28		28	kappa = 0.95 (p<0.001)	92.9		93.9

* McNemar Test χ^2^ : p<0.05 for the comparison of all categorical variables.

Regarding the agreement between dentists and CHA on the prioritization of care for the evaluated people, the agreement was absolutely perfect in 41 of the 60 observed cases. On the other 19 cases, some disagreement were noted, however, in the majority of the cases they were minor ones (*e.g.,* in seven cases the dentists observed 3 or more cavities, while the CHA observed only 1 or 2 cavities; in two other cases, the dentists saw 1 or 2 dental decays while the CHA observed 3 or more cavities). Nevertheless, when the weighted kappa test was performed, a value of 0.71 was found.

## Discussion

The results show that the COHI can examine in a simplified and fast manner, as well as with sufficient sensitivity and specificity, the oral health problems common in the population. These characteristics allow its use in the planning process for the population oral health care.

According to the interpretation of Landis and Koch [13]–[14], a correlation can be classified as excellent (>.80), good (between.60 and.80), moderate (between.41 and.60), reasonable (between.21 and.40) and low (<.21). The results of this study showed excellent correlation and agreement between the COHI and DMFT when used by dentists. It is important to know that this type of correlation and agreement is desirable, considering that an excellent agreement in the correlation indicates that both indexes are measuring exactly the same phenomenon [15]. A weak correlation (<.21) between the two rates may indicate that the difference between them is too great not to be considered, and probably the indexes are measuring factors and/or different phenomena. When the COHI was used by dentists and CHA, the values of correlation and agreement ranged between excellent and moderate. When the variables studied were the number of teeth and residual roots, as well as the use and/or need of prosthesis the values were excellent, while values were moderate to good when evaluating the categorization of dental cavities and soft tissue lesions.

For the comparison of continuous COHI variables evaluated by dentists and CHA the Cohen's kappa test *(weighted Cohen's kappa)* was chosen due to its ability to validate data such as the one used in the research. Despite the amount of existing tests for assessing the relationship of variables, such as *kappa,* chi-square, etc., there is a limitation of instruments that can be used to compare continuous variables. The correlation tests (Spearman and Pearson) are limited by, as the name implies, performing only the correlation between variables and not the agreement between them. If, for example, one measure is always double the other in any systematic bias, there will be a perfect correlation between them, despite no agreement among them [16]. The weighted Cohen's kappa test [17], allows evaluating, differently, the disagreements between the examiners, where a difference of 1 point can be considered less incorrect than a difference of 2 points on the scale used. Although the weighted kappa typically estimate greater values than the simple kappa, this is not necessarily true when major differences (*e.g.,* 1–4) are more common than minor differences (*e.g.,* 1–2). Therefore, the use of correlation tests as well as the weighted *kappa* test strengthens the validation of the instrument.

One can also argue that 60 individuals are a small number of subjects for an instrument validation; however, this number was utilized based on the WHO guidelines [11]. Additionally, articles regarding validation with smaller numbers than those are common in the literature [18]–[24]. The fact that no statistical difference was found between CHA and DS when utilizing the COHI, corroborates to the fact that 60 individual were enough to validate this instrument. As this study was not an epidemiological one, the effort was to guarantee individuals that would simulate all range of problems evaluated in the new instrument, and not individuals that would represent their community. Nevertheless, it is believed by the authors that the individuals examined also represent the dental findings of their community. As a result of this effort, it can be found DMF-T ranging between 0 and 32, with a range of 0–28 for carious lesions, 0–32 for extracted teeth and 0–13 for restored ones. Additionally, 10% of the individuals presented some type of soft tissue lesion, 42,6% tartar, 19,7% gingival inflammation, and 90,2% dental cavities. These characteristics guarantee that the DS and CHA were exposed to a diverse set of realities.

Clinical examination was performed by dentists and CHA on the same day and time (with a difference of minutes) and under the same conditions (*e.g.*, light, instrumental, chair, position of the subject). This avoids the possible circumstantial differences between the examinations, such as different levels of oral hygiene and luminosity, and validates the findings of the research. The training and calibration of the dentists and CHA occurred separately, thus the different categories were submitted to these activities at different times. The choice of this division was to ensure that professionals feel less intimidated by the presence of another professional class, and might feel freer to ask their peers, as well as their training and calibration supervisors, questions, thus enabling better participation and enjoyment of them.

The advantage of this index compared to other previously validated in the literature and used in scientific research, is the use of other professionals, rather than dentists, for its implementation. In the Brazilian National Health System (SUS), the CHA is the professional responsible for the link between the community and the Health Care Unit, where traditionally the other health professionals are present, among them, the dentist. Thus, the use of CHA for the utilization of a simplified oral examination, which uses the COHI, is viable and has huge advantages. One can mention as an example the ease of implementation of this examination by the CHA due to its proximity and identification to the population. The economy of resources can also be cited, as the CHA labor is less costly. Additionally, there is greater flexibility and agility in the examinations, because the staff is already working in the area ascribed to the health care unit. All these factors allow greater populational coverage and better action planning. Nevertheless, it is important to know that the COHI can be used by other professionals, including those without graduate level – as the CHA.

Another advantage of the COHI over other oral health indexes is the fact that this is not limited to the evaluation of oral cavity hard tissues, as the DMFT. The COHI assess the need for prostheses and soft tissues injuries and disorders, which is essential for early detection and prevention of oral cancer.

The use of the COHI by other professionals instead of a dentist, provides more time, and enables the dentist to perform actions that only he/she can do (*e.g.,* curative care), or that are better developed in the presence of a dentist (*e.g.,* planning of preventive and community based actions in the oral health field). Additionally, the use of COHI contributes to the dental knowledge demystification, given to the co-responsibility between the health team members on health production and promotion. This new form of production contributes to the development of professional practice based on respect for the User identity, knowledge of the family context and work activities, allowing time to listen to the complaint and the care, utilizing the appropriate steps, creating supports for comprehensive health care and the needs of different population groups.

The COHI aims to assess population and individuals’ current oral health status, and, with this information, to be able to organize and prioritize care. As mentioned before, this index checks the masticatory capacity by counting the number of teeth, the need for curative treatment by counting the visible teeth with cavities and residual root, the presence of soft tissues injuries, and the use and need of dental prosthesis. Additionally, it possesses a list of clinical signs related to periodontal and dental cavities problems, as well as soft tissue injuries, which allows the prioritization of individuals in more severe need. Therefore, those measures contribute to the purpose of the instrument, and allow the health care team to organize and prioritize care based on population needs.

Despite the importance of this research, one cannot rule out the bias of those who participated in it (the sample was comprised of volunteers - a convenience sample), which may contribute to the achievement of stronger correlations than in the general population, since the volunteers can have different motivations to participate in the research. However, this issue is more important when volunteers need to respond to any questionnaire and/or give subjective information, or even in the case of a homogeneous group of volunteers, which was not what happened in this study. The data collected are objective (*e.g.,* number of teeth and residual roots in the mouth, tooth decay, use and need of prosthesis) and are not influenced by the subjectivity of the volunteers. Additionally, the volunteers had diversity in terms of sex, age and oral health condition, which resulted in the exposure of examiners to different realities.

Besides being valid and reliable, a good index should have a proper cost-benefit analysis, be easily implemented and well accepted by investigators and those being evaluated [24]. In spite of not being the main purpose of this study to assess these characteristics, there is confidence by the authors that the COHI has all these characteristics, as the index is easy to use and there was no complaint and/or discomfort by the examiners and those examined during its utilization.

It can be concluded that the COHI is a valid instrument to detect oral health problems and can be, after training, largely used by non-dental professionals. There is consciousness that some professionals may present health skepticism on an oral health index that is not performed by dentists; however, there is confidence that the simplicity, accuracy and usefulness of this index will supersede these initial concerns.
